# Information Geometry of *κ*-Exponential Families: Dually-Flat, Hessian and Legendre Structures

**DOI:** 10.3390/e20060436

**Published:** 2018-06-05

**Authors:** Antonio M. Scarfone, Hiroshi Matsuzoe, Tatsuaki Wada

**Affiliations:** 1 Istituto dei Sistemi Complessi, Consiglio Nazionale delle Ricerche (ISC-CNR), c/o Politecnico di Torino, 10129 Torino, Italy; 2 Department of Computer Science and Engineering, Graduate School of Engineering, Nagoya Institute of Technology, Gokiso-cho, Showa-ku, Nagoya 466-8555, Japan; 3 Region of Electrical and Electronic Systems Engineering, Ibaraki University, Nakanarusawa-cho, Hitachi 316-8511, Japan

**Keywords:** *κ*-generalized statistical mechanics, information geometry, dually-flat geometry, Hessian geometry, Legendre structure, divergence functions, 02.40.-k, 89.70.Cf, 05.90.+m

## Abstract

In this paper, we present a review of recent developments on the κ-deformed statistical mechanics in the framework of the information geometry. Three different geometric structures are introduced in the κ-formalism which are obtained starting from three, not equivalent, divergence functions, corresponding to the κ-deformed version of Kullback–Leibler, “Kerridge” and Brègman divergences. The first statistical manifold derived from the κ-Kullback–Leibler divergence form an invariant geometry with a positive curvature that vanishes in the κ→0 limit. The other two statistical manifolds are related to each other by means of a scaling transform and are both dually-flat. They have a dualistic Hessian structure endowed by a deformed Fisher metric and an affine connection that are consistent with a statistical scalar product based on the κ-escort expectation. These flat geometries admit dual potentials corresponding to the thermodynamic Massieu and entropy functions that induce a Legendre structure of κ-thermodynamics in the picture of the information geometry.

## 1. Introduction

In the study of complex systems, long tailed probability distributions are often discussed. Anomalous statistical behavior is largely observed in physical systems plagued by long-range interactions or long-time memory effects as well as in non-physical systems, such as biological, social and economic ones. In such systems, family of probability distributions characterized by deformed exponentials with asymptotic power-law tails play a relevant role.

To deal with these new phenomenologies, in recent decades, a generalized version of statistical mechanics has been formulated where power-law distributions are expressed in terms of a generalized exponential function which maximizes suitable entropic forms.

In recent years, there is a growing interest in studying statistical physics, including its generalized versions, by means of information geometry [1,2]. In this framework, the methods of the differential geometry are applied to study the properties of statistical manifolds constructed starting from a given parameterized probability distribution function.

The usefulness of the geometric approach in the study of the foundations of statistical mechanics and thermodynamics has been clear since from the early works of Gibbs and Charathéodory [3,4].

On the thermodynamical ground, after the pioneering works of Ruppeiner (see [5] for a review), which introduces a Riemannian metric in the thermodynamical parameter space starting from potentials like entropy and free energy, a rich literature has been developed to study the implications that geometry might have on the thermostatistics and in particular about its contact structure [6,7], the relationships between physical observable [8,9] and the thermodynamics transformations in a physical system [10,11].

Otherwise, the information geometry gives priority to the probabilities distributions. In this framework, one investigates the geometric structures of the statistical manifold generated by parametric families of distributions, by introducing a Riemann metric in the probability distribution space [12], identified with the Fisher metric [13]. After that, many efforts have been done to better understand the role of geometry in statistics, like, to cite a few, Čencov [14], that introduced the affine connection in a statistical manifold, Csizsár [15], that introduced the concept of *f*-divergence, Efron [16], that studied the role of curvature in a statistical model, Eguchi [17] that relates the affine connection to a divergence function or relative entropy and Amari [18,19,20], that further developed the differential geometry of statistical models by elucidating its dualistic nature and introducing the Pythagorean theorem and the projective theorem in the framework of information geometry.

More recently, Amari and co-workers showed the existence of a rich geometry in the probability space, the so-called α-geometry, consisting of a dually affine connection endowed by a Hessian structure [21], that is pertinent, in particular, to study of deformed exponential family [22,23,24,25].

In recent years, a certain interest has been given to the study of some probability distributions generated by deformed exponential families in which the exponential function is replaced by its generalized version. For instance, in [26] a family of generalized exponentials has been introduced by means of a positive increasing function and the corresponding thermostatistics was studied. Belonging to this family, the κ-exponential [27,28], at the heart of the κ-deformed statistical mechanics introduced by Kaniadakis in [29,30], is useful to study anomalous systems characterized by non-Gibbsian distributions with an asymptotic free-scale behavior.

Up today, many papers have been written on the foundations and the theoretical consistency of κ-statistical mechanics [31,32,33,34,35,36,37,38,39,40,41,42,43,44,45,46,47,48] (see also [49] and references therein). It has been applied in many research fields, such as statistical physics, thermostatistics, financial physics, social science, statistics and information theory [50,51,52,53,54,55,56,57,58,59,60,61,62,63,64,65].

In this paper, inspired by a recent work of Zhang and Naudts [66,67], we study the κ-distribution and its associated statistical manifold in the framework of information geometry, revisiting some results already known [68,69,70] and deriving new geometrical structures obtained from three different, not equivalent, divergence functions corresponding to the κ-deformed version of Brègman, “Kerridge” and Kullback–Leibler divergences.

It is known that starting from a deformed exponential family, one can introduce several kinds of different statistical manifold [71,72,73]. These can be derived from a hierarchical structure of escort expectations [74,75]. In particular, a Fisher metric can be obtained by introducing an expectation based on the first order escort distribution while a cubic form can be obtained by introducing an expectation based on the second escort distribution.

Escort averages can be useful to overcome several problems plaguing power-law distributions. For instance, such distributions have not finite momenta of any order as in κ-statistics where $\langle x^{n}\rangle <\infty$ only for n<1/κ−1 [76]. An expectation defined by means of escort distribution might be more appropriate to solve these and other questions, although other types of nonlinear expectation can also be introduced [77].

In the framework of κ-deformed formalism escort distributions has been previously introduced in [68] to investigate a dually flat geometry in the κ-distribution space and than a double escort distribution has been defined in [70] to study a second dually flat geometry, which is based on the escort expectation instead of the standard expectation. In both these geometric structures a kind of fluctuation-response relation, that could be relevant in the framework of the non-equilibrium κ-deformed statistical mechanics, has been deduced by using, respectively, escort and double escort expectations. In addition, escort distribution [78] appears recurrently in the research area of multi-fractals and generalized statistical mechanics.

In this paper, we show that within the κ-deformed exponential formalism, one can introduce three kinds of geometrical structures. The first, is an invariant geometry where the Fisher information is the unique Riemannian metric together with a dual pair of invariant affine connections. The other two, are dually-flat geometries that introduce a Legendre structure and a Hessian structure on the corresponding statistical manifold [70] where some potential functions, like the Massieu function $\psi_{\kappa }$ and the entropic form $S_{\kappa }$, are related in a way formally similar to what is done in the Kählerian geometry.

The main novelty of this work is to illuminate on the existent of an invariant geometry related to the κ-deformed formalism that has been never explored in the existent literature as well as on the existent of another dually-flat structure different from, but related to the one already studied in [70] that is characterized by scaled potentials, named para-potentials, and by a slimmer expression for the probability distribution.

The structure of the paper is as follows: In the next Section 2 we summarize the main aspect of some κ-deformed functions given by $\exp_{\kappa }\left(x\right)$, $\ln_{\kappa }\left(x\right)$ and $u_{\kappa }\left(x\right)$, that represent the bricks constituting the entire κ-deformed formalism. In Section 3, for the convenience of the reader, we present an overview of the (now) classical information geometry. The three statistical manifolds derived from different version of divergence function are studied in Section 4 and in particular, in Section 4.1 we introduce the statistical manifold derived from the κ-Kullback–Leibler divergence that is an invariant geometry with a positive curvature, while in Section 4.2 and Section 4.3 we introduced two dually-flat geometries with their Hessian structures obtained, respectively, from the κ-Kerridge and κ-Brègman divergences. Our conclusions are reported in Section 5.

## 2. κ-Deformed Functions

To begin with, let us review some preliminary facts concerning the κ-deformed functions remanding to the relevant literature for the details [26,27,37].

Firstly, the κ-logarithm, defined in

(1) $$\begin{array}{c} \ln_{\kappa }\left(x\right)=\frac{x^{\kappa }-x^{-\kappa }}{2\;\kappa }\;, \end{array}$$

where, for $\kappa^{2}<1$, $\ln_{\kappa }\left(x\right)=\ln_{-\kappa }\left(x\right)$ is a continuous, monotonic, increasing and concave function for $x\in R^{+}$, with $\ln_{\kappa }\left(R^{+}\right)\subseteq R$. It fulfills the relation $\lambda \;\ln_{\kappa }\left(\varepsilon \right)=1$ that will be useful in the remainder, with $\varepsilon ={\left(\frac{1+\kappa }{1-\kappa }\right)}^{1/2\;\kappa }$ and $\lambda =\sqrt{1+\kappa^{2}}$. In the κ→0 limit, these constants recover the Neperian natural number and the unity, i.e., ε=e and λ=1, as well as, in the same limit, the κ-logarithm reduces to the standard logarithm $\ln_{0}\left(x\right)=\ln\left(x\right)$. Remarkably, $\ln_{\kappa }\left(x\right)$ satisfies the relation $\ln_{\kappa }\left(x\right)=-\ln_{\kappa }\left(1/x\right)$ like does the standard logarithm.

Another κ-deformed function recurrent in this formalism, namely *u*-function, is given by

(2) $$\begin{array}{c} u_{\kappa }\left(x\right)=\frac{x^{\kappa }+x^{-\kappa }}{2}\;. \end{array}$$

For $\kappa^{2}<1$, the *u*-function $u_{\kappa }\left(x\right)=u_{-\kappa }\left(x\right)$ is continuous for $x\in R^{+}$, with $u_{\kappa }\left(R^{+}\right)\subset R^{+}$, $u_{\kappa }\left(0\right)=u_{\kappa }\left(+\infty \right)=+\infty$ and obtains its minimum at x=1.

Like $\ln_{\kappa }\left(x\right)$, the deformed function $u_{\kappa }\left(x\right)$ satisfies the same relations $\lambda \;u_{\kappa }\left(\varepsilon \right)=1$ as well as $u_{\kappa }\left(x\right)=u_{\kappa }\left(\frac{1}{x}\right)$ while it becomes a constant in the κ→0 limit: $u_{0}\left(x\right)=1$.

The two functions $\ln_{\kappa }\left(x\right)$ and $u_{\kappa }\left(x\right)$ are closely related each other in several ways. For instance, from the calculus we have

(3) $$\begin{array}{c} \frac{d}{dx}\ln_{\kappa }\left(x\right)=\frac{u_{\kappa }\left(x\right)}{x}\;,\;\;\frac{d}{dx}u_{\kappa }\left(x\right)=\kappa^{2}\;\frac{\ln_{\kappa }\left(x\right)}{x}\;. \end{array}$$

These two relations can be rewritten in an integral form according to

(4) $$\begin{array}{c} \ln_{\kappa }\left(x\right)=\int_{1}^{x}\frac{u_{\kappa }\left(y\right)}{y}\;dy\;,\;\;u_{\kappa }\left(x\right)=\kappa^{2}\int_{1}^{x}\frac{\ln_{\kappa }\left(y\right)}{y}\;dy+1\;, \end{array}$$

that can be assumed as the defining relations for these κ-deformed functions.

A very general approach to introduce deformed logarithms using an integral relation has been formulated in [26]. Let ϕ(s) be a strictly increasing function, we can define a deformed logarithm, namely ϕ-logarithm, in

(5) $$\begin{array}{c} \ln_{\phi }\left(x\right)=\int_{1}^{x}\frac{1}{\phi (s)}\;ds\;. \end{array}$$

When we choose ϕ(s)=s, the ϕ-logarithm reduces to the standard logarithm ln(x). Otherwise, by posing $\phi \left(s\right)=s/u_{\kappa }\left(s\right)$, Equation (5) is just the first of Equation (4), and the ϕ-logarithm reproduces the κ-logarithm (1).

It is interesting to note that, according to Equation (4), the function $u_{\kappa }\left(x\right)$ can be introduced as like as the κ-logarithm, by posing $\phi \left(s\right)=s/(\kappa^{2}\;\ln_{\kappa }\left(s\right))$. However, it should be noted that $u_{\kappa }\left(x\right)$ is not a deformed logarithm since $s/(\kappa^{2}\;\ln_{\kappa }\left(s\right))$ is not a monotonic increasing function.

The ϕ-logarithm admits the inverse function, namely the ϕ-exponential, defined in an implicit form as

(6) $$\begin{array}{c} \exp_{\phi }\left(x\right)=1+\int_{0}^{x}\phi \left(\exp_{\phi }\left(s\right)\right)\;ds\;. \end{array}$$

In the κ-formalism, this equation can be solved explicitly so that the κ-deformed exponential takes the expression

(7) $$\begin{array}{c} \exp_{\kappa }\left(x\right)={\left(\kappa \;x+\sqrt{1+\kappa^{2}\;x^{2}}\right)}^{1/\kappa }\;. \end{array}$$

Its analytic properties follows from those of the κ-logarithm, i.e., for $\kappa^{2}<1$, $\exp_{\kappa }\left(x\right)=\exp_{-\kappa }\left(x\right)$ is a continuous, monotonic, increasing and convex function, with $\exp_{\kappa }\left(R\right)\subseteq R^{+}$ and $\exp_{\kappa }\left(0\right)=1$. Moreover, it satisfies the relation $\exp_{\kappa }\left(-x\right)\;\exp_{\kappa }\left(x\right)=1$ like the standard exponential does.

On the physical ground, equilibrium distribution of a system described by an entropic form S(p) constrained by a given observable *U*, for instance, the internal energy, can be obtained from the optimal problem

(8) $$\begin{array}{c} {\mathrm{Max}}_{p}\;\left(S\left(p\right)-\beta \;E_{p}\left[\epsilon \right]\right)=0\;, \end{array}$$

where β is the Lagrange multipliers associated to the constraint $U=E_{p}\left[\epsilon \right]$, with $E_{p}\left[x\right]=\sum_{\mu }p_{\mu }\;x^{\mu }$ the standard linear average and $\epsilon^{\mu }$ are the outcomes of the observable corresponding, for instance, to the available energy levels of the system (hereinafter, Greek indices run on 0 to *n*; Latin indices run on 1 to *n*).

In Equation (8) we assumed, at priori, the normalization of pdf so that, we consider $p_{0}=1-\sum_{i}p_{i}$ as a function of the other $p_{i}$ avoiding to introduce, in this way, the normalization as a further constraint. In the κ-formalism, entropy assumes the following trace-form expression [29,30]

(9) $$\begin{array}{c} S_{\kappa }\left(p\right)=-\sum_{\nu }p_{\nu }\;\ln_{\kappa }\left(p_{\nu }\right)\equiv -E_{p}\left[\ln_{\kappa }\left(p\right)\right]\;, \end{array}$$

which reduces to the Boltzmann–Gibbs–Shannon entropy $S^{\mathrm{BGS}}\left(p\right)=-\sum_{\mu }p_{\mu }\;\ln\left(p_{\mu }\right)$ in the κ→0 limit. The optimal problem (8) becomes

(10) $$\begin{array}{c} \frac{\delta }{\delta p_{i}}\;\left\{-\left(1-\sum_{i}p_{i}\right)\;\ln_{\kappa }\left(1-\sum_{i}p_{i}\right)-\sum_{i}p_{i}\;\ln_{\kappa }\left(p_{i}\right)-\beta \;\left[\epsilon^{0}\;\left(1-\sum_{i}p_{i}\right)+\sum_{i}\epsilon^{i}\;p_{i}\right]\right\}=0\;, \end{array}$$

that, solved for $p_{i}$, gives

(11) $$\begin{array}{c} p_{i}=\frac{1}{\varepsilon }\;\exp_{\kappa }\left(\frac{1}{\lambda }\;\left(\theta^{i}-\gamma (\theta \right)\right)\;, \end{array}$$

where we have posed $\theta \equiv \{\theta^{i}\}$, with

(12) $$\begin{array}{c} \theta^{i}=-\beta \;\left(\epsilon^{i}-\epsilon^{0}\right)\;,\;\mathrm{and}\;\gamma \left(\theta \right)=-\lambda \;\ln_{\kappa }\left(\varepsilon \;p_{0}\right)\;. \end{array}$$

Here, γ(θ) is a function of parameters $\theta^{i}$ and is fixed by the normalization condition.

Strictly related to a given entropic form, the divergence function plays a relevant role in information theory. In a sense, it measures the “distance” between an arbitrary distribution $p_{\mu }$ and the reference distribution $q_{\mu }$, although it is not rigorously a “distance” function since, in general, is not symmetric in its arguments and does not satisfy the triangle inequality.

Within the Shannon entropy, the Kullback–Leibler divergence D[p;q] [79] is defined as

(13) $$\begin{array}{c} D\left[p;\;q\right]=\sum_{\mu }p_{\mu }\;\ln\left(\frac{p_{\mu }}{q_{\mu }}\right)\;, \end{array}$$

and can be written in the equivalent forms

(14) $$\begin{array}{c} D\left[p/\;q\right]=\sum_{\mu }p_{\mu }\;\left(\ln\left(p_{\mu }\right)-\ln\left(q_{\mu }\right)\right)\;, \end{array}$$

that we will call, with abuse of language, “Kerridge” divergency, because the cross term is known in information theory as Kerridge inaccuracy [80], or also in

(15) $$\begin{array}{c} D\left[p∥q\right]=S\left[q\right]-S\left[p\right]+\sum_{\mu }\frac{\partial S[q]}{\partial q_{\mu }}\;\left(p_{\mu }-q_{\mu }\right)\;, \end{array}$$

where, this last expression is known as Brègman divergence [81].

However, the situation is more complicated in the κ-formalism [44] since, in this case, the above expressions, rewritten in

(16) $$\begin{array}{ccc} D_{\kappa }\left[p;\;q\right] & = & \sum_{\mu }p_{\mu }\;\ln_{\kappa }\left(\frac{p_{\mu }}{q_{\mu }}\right)\;, \end{array}$$

(17) $$\begin{array}{ccc} D_{\kappa }\left[p/\;q\right] & = & \sum_{\mu }p_{\mu }\;\left(\ln_{\kappa }\left(p_{\mu }\right)-\ln_{\kappa }\left(q_{\mu }\right)\right)\;, \end{array}$$

(18) $$\begin{array}{ccc} D_{\kappa }\left[p∥q\right] & = & S_{\kappa }\left[q\right]-S_{\kappa }\left[p\right]-\lambda \sum_{\mu }\left(p_{\mu }-q_{\mu }\right)\ln_{\kappa }\left(\varepsilon \;q_{\mu }\right)\;, \end{array}$$

turn out to be no more equivalent, nor related to each other in a simple manner, as in the κ=0 case.

Functionals (16)–(18) are non-negative definite and vanish iff p=q, a property that follows from the concavity of $\ln_{\kappa }\left(x\right)$. In particular, the κ-Brègman divergence (18) has been introduced and studied in [37].

## 3. Statistical Manifold and Its Hessian Structure

In this section, we summarize the main aspects of a statistical manifold [1,2].

Let $\theta \equiv \{\theta^{1},\ldots ,\;\theta^{m}\}\in \Xi$ a set of *m* real parameters, where Ξ is an open subset of $R^{m}$. We introduce a discrete statistical model S, the (n+1)-dimensional simplex of discrete probability functions on the simple space Ω:

(19) $$\begin{array}{c} S=\left\{p\equiv \left\{p_{\mu }\left(\theta \right)\right\}:\;\Omega \to R\;\;|\sum_{\mu }p_{\mu }\left(\theta \right)=1,\;p_{\mu }\left(\theta \right)>0\right\}\;, \end{array}$$

where μ=0,…,n, labels the n+1 possible outcomes with probability $p_{\mu }$.

Under suitable conditions S can be regarded as a manifold with local coordinates system θ, endowed by a metric tensor

(20) $$\begin{array}{c} g_{ij}=E_{p}\left[\partial_{i}\ell \;\partial_{j}\ell \right]\;, \end{array}$$

with $\partial_{i}=\partial /\partial \theta^{i}$ and $\ell \equiv \left\{\ell_{\nu }\left(\theta \right)\right\}=\left\{\ln p_{\nu }\left(\theta \right)\right\}$, by a cubic form

(21) $$\begin{array}{c} C_{ijl}=E_{p}\left[\partial_{i}\ell \;\partial_{j}\ell \;\partial_{l}\ell \right]\;, \end{array}$$

that is a third-order completely symmetric tensor and by a Riemannian (Levi-Civita) connection

(22) $$\begin{array}{c} \Gamma_{ij,l}^{\left(0\right)}=\frac{1}{2}\left(\partial_{i}g_{jl}+\partial_{j}g_{il}-\partial_{l}g_{ij}\right)\;. \end{array}$$

Starting from definitions (21)–(22) we can introduce a family of affine connections, named α-connection $\nabla^{\left(\alpha \right)}$, with α∈R, defined in

(23) $$\begin{array}{c} \Gamma_{ij,l}^{\left(\alpha \right)}=\Gamma_{ij,l}^{\left(0\right)}-\frac{\alpha }{2}\;C_{ijl}\;. \end{array}$$

In particular, 1-connection and −1-connection play a special role in information geometry. They are called, respectively, exponential-connection $\nabla^{\left(e\right)}\equiv \nabla^{\left(1\right)}$, and mixture-connection $\nabla^{\left(m\right)}\equiv \nabla^{\left(-1\right)}$ and in a local representation are given by

(24) $$\begin{array}{c} \Gamma_{ij,l}^{\left(1\right)}=E_{p}\left[\partial_{ij}\ell \;\partial_{l}\ell \right]\;,\;\;\Gamma_{ij,l}^{\left(-1\right)}=E_{p}\left[\frac{1}{p}\partial_{ij}p\;\partial_{l}\ell \right]\;. \end{array}$$

We recall that metric (20) is known in statistics as Fisher information and is the only Riemannian metric invariant under sufficient statistics on the manifold of a probability distribution. For this reason, the geometry defined by Equations (20)–(22) is called invariant geometry.

More in general, starting from the affine connection ∇, we can introduce with respect to *g* a dual connection $\nabla^{*}$, defined by of the relation

(25) $$\begin{array}{c} \partial_{l}g_{ij}=\Gamma_{li,j}+\Gamma_{lj,i}^{*}\;. \end{array}$$

We say the triplet (Ξ,g,∇) is a statistical manifold if ∇g is totally symmetric, that is

(26) $$\begin{array}{c} \partial_{j}g_{il}-\Gamma_{ji,l}-\Gamma_{jl,i}=\partial_{i}g_{jl}-\Gamma_{ij,l}-\Gamma_{il,j}\;, \end{array}$$

and in addition, if *g* is torsion-free, also $\nabla^{*}g$ is totally symmetric. Therefore, $\left(\Xi ,\;g,\;\nabla^{*}\right)$ is again a statistical manifold dual to (Ξ,g,∇).

We say the statistical manifold (Ξ,g,∇) is flat if ∇ is curvature-free. In this case, there exist locally on Ξ a coordinate systems θ, named affine coordinate systems, such that the connection $\Gamma_{ij,l}$ vanish on its coordinates neighbourhood.

It can be shown that $\Gamma^{\left(\alpha \right)}$ and $\Gamma^{\left(-\alpha \right)}$ are dual to each other and this hold also for the exponential and the mixture connection. In particular, as it is well-known, the statistical model defined by the exponential family

(27) $$\begin{array}{c} S^{\exp}=\left\{p\equiv \left\{p_{\mu }\left(\theta \right)\right\}:\;\Omega \to R\;\;|\;p_{i}\left(\theta \right)=\exp\left[\sum_{j}\theta^{j}\;c_{j}\left(x_{i}\right)-\gamma \left(\theta \right)\right]\;,\;p_{0}\left(\theta \right)=1-\sum_{i}p_{i}\left(\theta \right)\right\}\;, \end{array}$$

where $c_{j}\left(\chi \right)$ are given functions of a random variable χ and γ(θ) is the normalization factor, admits a set of affine coordinates such that the exponential connection vanishes and the corresponding statistical manifold is said exponential-flat. In the same way, the statistical model defined by the mixture family

(28) $$\begin{array}{c} S^{\mathrm{mix}}=\left\{p\equiv \left\{p_{\mu }\left(\eta \right)\right\}:\;\Omega \to R\;\;|\;p_{i}\left(\eta \right)=\left(1-\sum_{j}\eta_{i}\right)\;r_{0}\left(x_{i}\right)+\sum_{j}\eta_{j}\;r_{j}\left(x_{i}\right),\;p_{0}\left(\eta \right)=1-\sum_{i}p_{i}\left(\eta \right)\right\}\;, \end{array}$$

where $r\left(\chi \right)=\{r_{\mu }\left(\chi \right)\}$ are given distribution functions of a random variable χ, with $\sum_{\mu }\eta_{\mu }=1$, admits a set of affine coordinates such that the mixture connection vanishes and the corresponding statistical manifold is said mixture flat.

If ∇ is flat on Ξ, we say that the statistical manifold (Ξ,g,∇) form a Hessian structure on Ξ if there exists a function ψ≡ψ(θ) such that, at least locally, the following formula holds

(29) $$\begin{array}{c} g_{ij}\left(\theta \right)=\partial_{ij}\psi \;. \end{array}$$

In this case, be θ a ∇-affine coordinate system on Ξ, there exist a dual ∇-affine coordinate system $\eta \equiv \{\eta_{j}\}$ on the dual space Ξ, such that

(30) $$\begin{array}{c} g\left(\partial_{i},\;\partial^{j}\right)=\delta_{i}^{j}\;, \end{array}$$

where $\partial^{j}=\partial /\partial \eta_{j}$ and in addition, the following relation hold

(31) $$\begin{array}{c} g^{ij}\left(\eta \right)=\partial^{ij}\varphi \;, \end{array}$$

for some function φ, where $g^{ij}$ is the inverse of the Riemannian metric $g_{ij}$, with

(32) $$g^{il}\;g_{lj}=\delta_{j}^{i}\;,\;\;g^{ij}=\partial^{i}\theta^{j}\;,\;\;g_{ij}=\partial_{i}\eta_{j}\;,$$

while the cubic form is given by

(33) $$\begin{array}{c} C_{ijl}=\partial_{ijl}\psi \;,\;\mathrm{and}\;C^{ijl}=\partial^{ijl}\varphi \;. \end{array}$$

It can be shown that the two dual-affine coordinate systems can be introduced according to

(34) $$\begin{array}{c} \eta_{i}=\partial_{i}\psi \;,\;\;\theta^{i}=\partial^{i}\varphi \;, \end{array}$$

where the two functions, named respectively ψ-potential and φ-potential, are related by the relation

(35) $$\begin{array}{c} \psi \left(\theta \right)+\varphi \left(\eta \right)-\sum_{i}\theta^{i}\;\eta_{i}=0\;, \end{array}$$

that introduces a Legendre structure on the statistical manifold.

Finally, let us define on Ξ×Ξ the function

(36) $$\begin{array}{c} D\left[p,\;q\right]=\psi \left(\theta (p)\right)+\varphi \left(\eta (q)\right)-\sum_{i}\theta^{i}\left(p\right)\;\eta_{i}\left(q\right)\;, \end{array}$$

called canonical divergence on the Hessian manifold (Ξ,g,∇), that is a Brégman divergence [81]. It introduces a pseudo-distance that is asymmetric, with D[p,q]≥0, where equality holds iff p=q, in agreement with Equation (35).

If (Ξ,g,∇) form a Hessian structure, it is easy to verify the following relations

(37) $$\begin{array}{c} \partial_{p^{i}}D\left[p,\;q\right]{|}_{p=q}=0\;,\;\;\;\partial_{p_{i}}D\left[p,\;q\right]{|}_{p=q}=0\;, \end{array}$$

(38) $$\begin{array}{c} \partial_{p^{i}q^{j}}D\left[p,\;q\right]{|}_{p=q}=-g_{ij}\;,\;\;\partial_{p_{i}q_{j}}D\left[p,\;q\right]{|}_{p=q}=-g^{ij}\;, \end{array}$$

(39) $$\begin{array}{c} \partial_{p^{i}p^{j}q^{l}}D\left[p,\;q\right]{|}_{p=q}=-C_{ijl}\;,\;\;\partial_{p_{i}p_{j}q_{l}}D\left[p,\;q\right]{|}_{p=q}=-C^{ijl}\;, \end{array}$$

(40) $$\begin{array}{c} \partial_{p^{i}q^{j}q^{l}}D\left[p,\;q\right]{|}_{p=q}=0\;,\;\;\;\partial_{p_{i}q_{j}q_{l}}D\left[p,\;q\right]{|}_{p=q}=0\;, \end{array}$$

where $\partial_{p^{i}}\equiv \partial /\partial \theta^{i}\left(p\right)$, $\partial_{p_{i}}\equiv \partial /\partial \eta_{i}\left(p\right)$ and similarly for ***q***.

More in general, following [82], any definite positive contrast function ρ[p,q], that is a divergence compatible with the structure of the statistical manifold, induces a Riemannian geometry on Ξ, not necessarily flat, whose metric and connections are given by

(41) $$\begin{array}{c} \partial_{p_{i}q_{j}}\rho \left[p,\;q\right]{|}_{p=q}=-g^{ij}\;,\;\partial_{p_{i}p_{j}q_{l}}\rho \left[p,\;q\right]{|}_{p=q}=-\Gamma^{ij,l}\;,\;\partial_{p_{l}q_{i}q_{j}}\rho \left[p,\;q\right]{|}_{p=q}=-\Gamma^{*\;ij,l}\;, \end{array}$$

and similarly for the low indices.

Remarkably, different contrast functions that share the same cross term induce the same geometric structure, since, according to the above equations, the crossing derivative vanishes for terms depending only on ***p*** or ***q***.

## 4. Statistical Manifolds in κ-Formalism

As known, any discrete distribution is in an exponential family and in a mixture family at the same time [2]. In fact, starting from the statistical model (19) we can define the distribution

(42) $$\begin{array}{c} p_{\nu }\left(\theta \right)=\sum_{\mu }p_{\mu }\;\vartheta_{\mu }\left(\nu \right)=\left(1-\sum_{i}p_{i}\right)\;\vartheta_{0}\left(\nu \right)+\sum_{i}p_{i}\;\vartheta_{i}\left(\nu \right)\;, \end{array}$$

with

(43) $$\begin{array}{c} \vartheta_{0}\left(\nu \right)=1-\sum_{i}\vartheta_{i}\left(\nu \right)\;, \end{array}$$

where $\vartheta_{i}\left(\nu \right)=1$ when the discrete random variable ν=i and zero otherwise, that clearly belongs to the mixture-family (28), with $r_{\mu }\left(x_{\nu }\right)\equiv \vartheta_{\mu }\left(\nu \right)$ and $\eta_{i}\equiv p_{i}$.

Otherwise, by applying the logarithm to Equation (42) we obtain the distribution

(44) $$\begin{array}{c} p_{\nu }\left(\theta \right)=\exp\left(\sum_{i}\left(\ln\left(p_{i}\right)-\ln\left(p_{0}\right)\right)\;\vartheta_{i}\left(\nu \right)+\ln\left(p_{0}\right)\right)\;, \end{array}$$

that belongs to the exponential-family (27), with $\theta^{\nu }\equiv \sum_{i}\left(\ln\left(p_{i}\right)-\ln\left(p_{0}\right)\right)$, $c_{\nu }\left(x_{i}\right)=\vartheta_{i}\left(\nu \right)$ and $\gamma \left(\theta \right)\equiv -\ln\left(p_{0}\right)$.

More in general, given a generalized logarithm function $\ln_{\phi }\left(x\right)$, from Equation (42) we can introduce the distribution

(45) $$\begin{array}{c} p_{\nu }\left(\theta \right)=\exp_{\phi }\left(\sum_{i}\left(\ln_{\phi }\left(p_{i}\right)-\ln_{\phi }\left(p_{0}\right)\right)\;\vartheta_{i}\left(\nu \right)+\ln_{\phi }\left(p_{0}\right)\right)\;, \end{array}$$

that is in a discrete generalized exponential-family with parameters $\theta^{\nu }\equiv \sum_{i}\left(\ln_{\phi }\left(p_{i}\right)-\ln_{\phi }\left(p_{0}\right)\right)$, $c_{\nu }\left(x_{i}\right)=\vartheta_{i}\left(\nu \right)$ and $\gamma \left(\theta \right)\equiv -\ln_{\phi }\left(p_{0}\right)$.

Therefore, the manifold of a discrete probability distribution is a super-manifold in which any statistical model of a discrete random variable, including ϕ-exponential family *a là Naudts*, is embedded as a submanifold. Different embedding of the same distribution induces different geometric structures which are related to non equivalent divergence functionals. In the following, we explore these alternatives starting from the divergence functionals introduced in Equations (16)–(18).

It is worth noticing that in the above arguments no hypothesis has been done on the origin of distribution ***p***. However, if this distribution came from an optimization problem, as in Equation (10), the coordinates $\theta^{i}$ would coincide with those introduced in Equation (12) and are related to the Lagrange multipliers of the corresponding constraints.

### 4.1. First Statistical Structure

The first geometric structure we are introducing came from the κ-deformed version of Kullback–Leibler divergence (16). It belongs to the family of the *f*-divergence studied by Csiszár [15]

(46) $$\begin{array}{c} D\left[p,\;q\right]=\sum_{\mu }p_{\mu }\;f\left(\frac{q_{\mu }}{p_{\mu }}\right)\;, \end{array}$$

where, in general, f(x) is any convex differentiable function satisfying the condition f(1)=0. Clearly, Equation (16) follows from (46) for $f\left(x\right)=-\ln_{\kappa }\left(x\right)$.

As known, any *f*-divergence induces a geometry invariant under sufficient statistics and, excepting the Kullback–Leibler divergence, the corresponding statistical manifold has not a Hessian structure nor is dually-flat and thence is characterized by a not vanishing curvature [2].

In this case, the embedding we are implementing is given by the standard logarithmic

(47) $$\begin{array}{c} p_{\mu }\to \ell_{\mu }=\ln\left(p_{\mu }\right)\;, \end{array}$$

where the natural coordinates are those of the exponential family $\eta \equiv \{p_{i}\}$ and $\theta \equiv \{\theta^{i}\}$.

By posing $\rho \left[p,\;q\right]\equiv D_{\kappa }\left[q;\;p\right]$ from Equations (41) we straightforward derive metric and connection in the Ξ space, according to

(48) $$\begin{array}{c} g^{ij}=\frac{1}{p_{0}}\;+\frac{\delta^{ij}}{p_{i}}\;, \end{array}$$

(49) $$\begin{array}{c} \Gamma^{ij,l}=\left(1+\kappa^{2}\right)\;\left(\frac{1}{p_{0}^{2}}-\frac{\delta^{ijl}}{p_{i}^{2}}\right)\;, \end{array}$$

(50) $$\begin{array}{c} \Gamma^{*\;ij,l}=-\kappa^{2}\;\left(\frac{1}{p_{0}^{2}}-\frac{\delta^{ijl}}{p_{i}^{2}}\right)\;, \end{array}$$

where $\delta^{ijl}=1$ for i=j=l and zero otherwise, and coincide, as expected, with the definitions of statistical Fisher information matrix and α-connection.

The inverse relations are given by

(51) $$\begin{array}{c} g_{ij}=p_{i}\;\left(\delta_{ij}-p_{j}\right)\;, \end{array}$$

(52) $$\begin{array}{c} \Gamma_{ij,l}=\left(1+\kappa^{2}\right)\;p_{i}\;\left[\left(\delta_{ijl}-p_{j}\;\delta_{jl}\right)-p_{j}\;\left(\delta_{il}-p_{l}\right)-p_{l}\;\left(\delta_{lj}-p_{j}\right)\right]\;, \end{array}$$

(53) $$\begin{array}{c} \Gamma_{ij,l}^{*}=-\kappa^{2}\;p_{i}\;\left[\left(\delta_{ijl}-p_{j}\;\delta_{jl}\right)-p_{j}\;\left(\delta_{il}-p_{l}\right)-p_{l}\;\left(\delta_{lj}-p_{j}\right)\right]\;, \end{array}$$

while from Equation (23) we can derive the Levi-Civita connection

(54) $$\begin{array}{c} \Gamma_{ij,l}^{\left(0\right)}=\frac{1}{2\;(1+\kappa^{2})}\;\Gamma_{ij,l}\;, \end{array}$$

that does not depend on the deformation parameter and the cubic form

$$\begin{array}{c} C_{ij,l}^{\left(\kappa \right)}=-\frac{1+2\;\kappa^{2}}{1+\kappa^{2}}\;\Gamma_{ij,l}\;. \end{array}$$

These relations confirm that κ-Kullback–Leibler divergence actually introduces the α-geometry in the framework of the κ-formalism where

(55) $$\begin{array}{c} \alpha =1+2\;\kappa^{2}\;, \end{array}$$

in agreement with the general relation $\alpha =3+2\;f^{\prime \prime \prime }\left(1\right)$ [23].

Remark that this correspondence holds only for α≥1, considering that the exponential family follows in the κ→0 limit. This should be compared with the results discussed in [25], where an invariant geometry has been derived in the framework of the Tsallis formalism of statistical mechanics starting from the corresponding relative entropy. In that case, the analogue relationships between parameters is given by α=1−2q, which is consistent for α<1.

The statistical manifold introduced in Equations (48)–(50) has not a dually flat structure, nor a Hessian structure, but it has a constant curvature whose Riemann-Christoffel tensor, given by

(56) $$\begin{array}{c} R_{ijls}^{\left(\kappa \right)}=\partial_{i}\Gamma_{jls}-\partial_{j}\Gamma_{ils}+\sum_{r}\left(\Gamma_{irs}\;\Gamma_{jl}^{\;\;r}-\Gamma_{jrs}\;\Gamma_{il}^{\;\;r}\right)\;, \end{array}$$

becomes

(57) $$\begin{array}{c} R_{ijls}^{\left(\kappa \right)}=c^{\left(\kappa \right)}\;\left(g_{jl}\;g_{is}-g_{il}\;g_{js}\right)\;, \end{array}$$

with

(58) $$\begin{array}{c} c^{\left(\kappa \right)}=\kappa^{2}\;\left(\kappa^{2}-1\right)\;. \end{array}$$

As expected, $R_{ijls}^{\left(\kappa \right)}$ vanishes in the κ→0 limit, where the whole geometric structure collapses to the dually-flat 1-geometry.

The Ricci curvature tensor and the scalar curvature are readily evaluated in

(59) $$\begin{array}{c} R_{ij}=\left(1-n\right)\;c^{\left(\kappa \right)}\;g_{ij}\;,\;\;R=\left(n-n^{2}\right)\;c^{\left(\kappa \right)}\;, \end{array}$$

that are positive definite quantities for $\kappa^{2}<1$.

Finally, in spite of the lack of an entropic form related to this invariant geometry, we can derive a particular family of discrete distributions within the κ-formalism adopting an optimization procedure based on the κ-deformed Kullback–Leibler divergence.

In this way, the equilibrium distribution achieving the minimum divergence of the function (16) is given by the following problem

(60) $$\begin{array}{c} \frac{\delta }{\delta p_{i}}\;\left\{\sum_{\mu }p_{\mu }\;\ln_{\kappa }\left(\frac{p_{\mu }}{q_{\mu }}\right)+\beta \;\sum_{\mu }\epsilon^{\mu }\;p_{\mu }\right\}=0\;, \end{array}$$

where we assume, at priori, the normalization for ***p***, that is $p_{0}=1-\sum_{i}p_{i}$, and similar for the target distribution ***q***. We get the κ-distribution in the form

(61) $$\begin{array}{c} p_{i}=\frac{q_{i}}{\varepsilon }\;\exp_{\kappa }\left(\frac{1}{\lambda }\;\left({\tilde{\theta }}^{i}-\gamma (\tilde{\theta }\right)\right)\;, \end{array}$$

with $\gamma \left(\tilde{\theta }\right)=-\lambda \ln_{\kappa }\left(\varepsilon \;p_{0}/q_{0}\right)$ and $\tilde{\theta }$, defined in a similar manner, as in Equation (12), are related to the natural coordinates θ in

(62) $$\begin{array}{c} \theta^{i}=\ln\left(\frac{q_{i}}{q_{0}}\right)+\frac{1}{\kappa }\left[\mathrm{arcsinh}\left(\frac{\kappa }{\lambda }\;\left({\tilde{\theta }}^{i}-\gamma \left(\tilde{\theta }\right)\right)\right)+\mathrm{arcsinh}\left(\frac{\kappa }{\lambda }\;\gamma \left(\tilde{\theta }\right)\right)\right]\;. \end{array}$$

Therefore, let ***q*** be a reference probability distribution on S, the sub-manifold of the statistical family

(63) $$\begin{array}{c} S_{\left(1\right)}^{\kappa }=\left\{p\equiv \left\{p_{\mu }\left(\tilde{\theta }\right)\right\}:\;\Omega \to R\;\;|p_{i}=\frac{q_{i}}{\varepsilon }\;\exp_{\kappa }\left(\frac{1}{\lambda }\left({\tilde{\theta }}^{i}-\gamma \left(\tilde{\theta }\right)\right)\right);\;p_{0}=1-\sum_{i}p_{i}\right\}\;, \end{array}$$

that optimize the divergence function (16) admits a Riemannian structure described by the Fisher metric (48), dual connection (49) and (50) and constant positive curvature (59). This geometric structure collapses, in the κ→0 limit, in the dually-flat geometry of the exponential distribution families.

### 4.2. Second Statistical Structure

The next geometric structure we are introducing came from κ-deformed version of “Kerridge” divergence (17). It is strictly related to the following statistical model

(64) $$\begin{array}{c} S_{\left(2\right)}^{\kappa }=\left\{p\equiv \left\{p_{\mu }\left(\theta \right)\right\}:\;\Omega \to R\;\;|p_{i}=\exp_{\kappa }\left(\theta^{i}-\gamma \left(\theta \right)\right);\;p_{0}=1-\sum_{i}p_{i}\right\}\;, \end{array}$$

consistent with the embedding

(65) $$\begin{array}{c} p_{\mu }\to \ln_{\kappa }\left(p_{\mu }\right)\;. \end{array}$$

To introduce a manifold structure on $S_{\left(2\right)}^{\kappa }$ we pose $\eta \equiv \{p_{i}\}$ so that, from relations (41) applied to $\rho \left[p,\;q\right]\equiv D_{\kappa }\left[q/\;p\right]$, we obtain metric and connection as

(66) $$\begin{array}{c} g^{ij}=\frac{u_{\kappa }\left(\eta_{0}\right)}{\eta_{0}}\;+\frac{u_{\kappa }\left(\eta_{i}\right)}{\eta_{i}}\;\delta^{ij}\;, \end{array}$$

(67) $$\begin{array}{c} \Gamma^{ij,l}=\lambda \;\frac{u_{\kappa }\left(\eta_{0}/\varepsilon \right)}{\eta_{0}^{2}}-\lambda \;\frac{u_{\kappa }\left(\eta_{i}/\varepsilon \right)}{\eta_{i}^{2}}\;\delta^{ijl}\;, \end{array}$$

(68) $$\begin{array}{c} \Gamma^{*\;ij,l}=0\;. \end{array}$$

These quantities define a dually-flat structure on $S_{\left(2\right)}^{\kappa }$ compatible with a Hessian geometry.

In fact, give the φ-potential as

(69) $$\begin{array}{c} \varphi \left(\eta \right)=-\frac{1}{\lambda^{2}}{\tilde{S}}_{\kappa }\left(\eta \right)\;, \end{array}$$

where ${\tilde{S}}_{\kappa }\left(\eta \right)$ is the parentropy introduced in [34] and related to the κ-entropy in

(70) $$\begin{array}{c} {\tilde{S}}_{\kappa }\left(\eta \right)=\lambda \;S_{\kappa }\left(\frac{\eta }{\varepsilon }\right)\;, \end{array}$$

metric and connection follows straightforward from Equations (31) and (33), respectively.

Equation (68) states that coordinate η is ∇-affine on Ξ, whereas its dual coordinate system is given by

(71) $$\begin{array}{c} \theta^{i}=\ln_{\kappa }\left(\eta_{i}\right)+\gamma \left(\theta \right)\;, \end{array}$$

with $\gamma \left(\theta \right)=-\ln_{\kappa }\left(\eta_{0}\right)$.

The ψ-potential can be obtained from the Legendre transform (35) on φ(η) and is given by

(72) $$\begin{array}{c} \psi \left(\theta \right)=\frac{1}{\lambda }\;{\tilde{I}}_{\kappa }\left(\theta \right)+\gamma \left(\theta \right)\;, \end{array}$$

where the pull-back of ${\tilde{I}}_{\kappa }\left(\theta \right)$ on Ξ, denoted in ${\tilde{I}}_{\kappa }^{*}\left(\eta \right)$, is defined in

(73) $$\begin{array}{c} {\tilde{I}}_{\kappa }^{*}\left(\eta \right)=\sum_{i}\eta_{i}\;u_{\kappa }\left(\frac{\eta }{\varepsilon }\right)\equiv E_{p}\left[u_{\kappa }\left(\frac{\eta }{\varepsilon }\right)\right]\;. \end{array}$$

Function ${\tilde{I}}_{\kappa }^{*}\left(\eta \right)$ is related to $I_{\kappa }^{*}\left(\eta \right)$, usually introduced in the κ-deformed statistical mechanics [35], according to

(74) $$\begin{array}{c} {\tilde{I}}_{\kappa }^{*}\left(\eta \right)=\lambda \;I_{\kappa }^{*}\left(\frac{\eta }{\varepsilon }\right)\;, \end{array}$$

where

(75) $$\begin{array}{c} I_{\kappa }^{*}\left(\eta \right)=\sum_{i}\eta_{i}\;u_{\kappa }\left(\eta \right)\equiv E_{p}\left[u_{\kappa }\left(\eta \right)\right]\;, \end{array}$$

reduces to unity in the κ→0 limit. In [34], functional $I_{\kappa }^{*}\left(\eta \right)$ has been related to the parentropy ${\tilde{S}}_{\kappa }\left[\eta \right]$, through the relation

(76) $$\begin{array}{c} I_{\kappa }^{*}\left(\eta \right)={\tilde{S}}_{\kappa }\left(\eta \right)-S_{\kappa }\left(\eta \right)+1\;. \end{array}$$

As will be highlighted in Section 4.3, ψ-potential is related on the physical ground to the κ-logarithm of partition function and to the scaled free energy, the para-free energy, of a physical system described by the parentropy (70), in agreement with the relation

(77) $$\begin{array}{c} {\tilde{F}}_{\kappa }=-\frac{1}{\beta }\left({\tilde{I}}_{\kappa }+\gamma \right)\;. \end{array}$$

By using the expression (72) we can verify the relation

(78) $$\begin{array}{c} \eta_{i}=\partial_{i}\left(\frac{1}{\lambda }\;{\tilde{I}}_{\kappa }^{*}\left(\frac{\eta }{\varepsilon }\right)+\gamma \left(\theta \right)\right)\;, \end{array}$$

so that metric and connections on the Ξ-space reads

(79) $$\begin{array}{c} g_{ij}=\frac{\eta_{i}}{u_{\kappa }\left(\eta_{i}\right)}\left(\delta_{ij}-\partial_{j}\gamma \right)\;, \end{array}$$

(80) $$\begin{array}{c} \Gamma_{ij,l}=0\;, \end{array}$$

(81) $$\begin{array}{c} \Gamma_{ij,l}^{*}=\frac{\eta_{i}}{u_{\kappa }\left(\eta_{i}\right)}\left[\lambda \;\frac{u_{\kappa }\left(\frac{\eta_{i}}{\varepsilon }\right)}{u_{\kappa }^{2}\left(\eta_{i}\right)}\left(\delta_{ij}-\partial_{i}\gamma \right)\;\left(\delta_{il}-\partial_{j}\gamma \right)-\partial_{jl}\gamma \right]\;, \end{array}$$

confirming the dually-flat structure of $S_{\left(2\right)}^{\kappa }$, with θ the ∇-affine coordinate on Ξ.

It is worthy to derive the canonical divergence for $S_{\left(2\right)}^{\kappa }$, that is a Brégman-like divergence obtainable from Equation (36). In fact, by using κ-parentropy it reads

(82) $$\begin{array}{c} D\left[p,\;q\right]=\frac{1}{\lambda^{2}}\left({\tilde{S}}_{\kappa }\left(q\right)-{\tilde{S}}_{\kappa }\left(p\right)\right)-\sum_{\mu }\left(p_{\mu }-q_{\mu }\right)\;\ln_{\kappa }\left(q_{\kappa }\right)\;, \end{array}$$

and, as expected, although differs from divergence (17), they share the same cross term so that both divergences (17) and (82) give rise to the same geometric structure.

Finally, it is easy to verify that exponential family $S_{\left(2\right)}^{\kappa }$ can be derived from the optimization problem (8), where the entropic form is given by the parentropy ${\tilde{S}}_{\kappa }\left(\eta \right)$ and the dual coordinate are related to the Lagrange multiplier according to Equation (12).

To go over in the study of this geometry, we show that the Hessian structure on Ξ is consistent with a scalar product on $S_{\left(2\right)}^{\kappa }$ induced by the κ-escort probability distribution

(83) $$\begin{array}{c} \langle A,\;B\rangle =E_{{\tilde{P}}^{\left(1\right)}}\left[A\;B\right]\;, \end{array}$$

where

(84) $$\begin{array}{c} E_{{\tilde{P}}^{\left(1\right)}}\left[f\right]=\sum_{\mu }{\tilde{P}}_{\mu }^{\left(1\right)}\;f^{\mu }\;, \end{array}$$

with

(85) $$\begin{array}{c} {\tilde{P}}_{\mu }^{\;(1)}=\frac{1}{{\tilde{U}}_{\kappa }^{\;\;(1)}}\frac{p_{\mu }}{u_{\kappa }\left(p_{\mu }\right)}\;,\;\mathrm{and}\;{\tilde{U}}_{\kappa }^{\;\;(1)}=\sum_{\mu }\frac{p_{\mu }}{u_{\kappa }\left(p_{\mu }\right)}\;. \end{array}$$

We employ the embedding

(86) $$\begin{array}{c} p_{\mu }\to {\tilde{\ell }}_{\mu }^{\left(\kappa \right)}=\ln_{\kappa }\left(p_{\mu }\right)\;, \end{array}$$

such that $E_{{\tilde{P}}^{\left(1\right)}}\left[\partial^{i}{\tilde{\ell }}^{\left(\kappa \right)}\right]=0$, which is our bias condition [69].

Introducing the tangent vector at point θ as

(87) $$\begin{array}{c} T^{j}=\partial^{j}{\tilde{\ell }}^{\;(\kappa )}\;, \end{array}$$

the metric follows from the standard definition

(88) $$\begin{array}{c} {\tilde{g}}^{\;ij}=\langle T^{i},\;T^{j}\rangle =\frac{1}{{\tilde{U}}_{\kappa }^{\;\;(1)}}\sum_{\mu }\frac{u_{\kappa }\left(p_{\mu }\right)}{p_{\mu }}\;\partial^{i}p_{\mu }\partial^{j}p_{\mu }\;, \end{array}$$

that is conformal equivalent to metric (66) according to [73]

(89) $$\begin{array}{c} {\tilde{g}}^{\;ij}=\frac{g^{ij}}{{\tilde{U}}_{\kappa }^{\;\;(1)}}\;. \end{array}$$

In the same manner, recalling that connection is related to the variation of the tangent vectors $T^{j}$ under infinitesimal changing of the parameters θ→θ+dθ, we consider the quantities $\partial^{i}T^{j}\equiv \partial^{ij}\;{\tilde{\ell }}^{\;(\kappa )}$, that projected on the base vectors $T^{l}$, gives

(90) $$\begin{array}{ccc} {\tilde{\Gamma }}^{ij,l} & = & \langle \partial^{i}T^{j},T^{l}\rangle =E_{{\tilde{P}}^{\left(1\right)}}\left[\partial^{ij}{\tilde{\ell }}^{\;(\kappa )}\;\partial^{l}{\tilde{\ell }}^{\;(\kappa )}\right]\;, \end{array}$$

which is related to Equation (67) by means of the conformal factor $U_{\kappa }^{\left(1\right)}$.

It is a duty to observe that $\partial^{i}T^{j}$ is actually unbiased because

(91) $$\begin{array}{c} E_{{\tilde{P}}^{\left(1\right)}}\left[\partial^{i}T^{j}\right]\neq 0\;, \end{array}$$

therefore the right way to obtain the connection elements is by biasing these quantities and then project them on the base vectors $T^{l}$ according to

(92) $$\begin{array}{ccc} {\tilde{\Gamma }}^{ij,l} & = & \langle \partial^{i}T^{j}-E_{{\tilde{P}}^{\left(1\right)}}\left[\partial^{i}T^{j}\right],T^{l}\rangle \;, \end{array}$$

that, nevertheless, reproduce the result (90) due to our bias condition.

Finally, Equation (68) follows from relation (25) and the results (66) and (67).

In a similar way, we can derive the expressions (79)–(81) from the definitions of $g_{ij}$, $\Gamma_{ij,l}$ and $\Gamma_{ij,l}^{*}$, that here rewrite in the κ-escort formalism

(93) $$\begin{array}{c} g_{ij}={\tilde{U}}_{\kappa }^{\;\;(1)}\;{\tilde{P}}_{i}^{\;(1)}\;\left(\delta_{ij}-{\tilde{P}}_{j}^{\;(1)}\right)\;, \end{array}$$

(94) $$\begin{array}{c} \Gamma_{ij,l}=0\;, \end{array}$$

(95) $$\begin{array}{c} \Gamma_{ij,l}^{*}={\tilde{U}}_{\kappa }^{\;\;(2)}\;{\tilde{P}}_{i}^{\;(2)}\;\left(\delta_{ij}-{\tilde{P}}_{j}^{\;(1)}\right)\;\left(\delta_{il}-{\tilde{P}}_{l}^{\;(1)}\right)-{\tilde{U}}_{\kappa }^{\;\;(1)}\;{\tilde{P}}_{i}^{\;(1)}\;\partial_{j}{\tilde{P}}_{l}^{\;(1)}\;, \end{array}$$

where we introduced the double-escort probability ${\tilde{P}}_{i}^{\left(2\right)}$ [70]

(96) $$\begin{array}{c} {\tilde{P}}_{i}^{\;\;(2)}=\frac{\lambda }{{\tilde{U}}_{\kappa }^{\;\;(2)}}\frac{p_{i}\;u_{\kappa }\left(p_{i}/\varepsilon \right)}{u_{\kappa }^{3}\left(p_{i}\right)}\;,\;\mathrm{with}\;{\tilde{U}}_{\kappa }^{\;\;(2)}=\lambda \sum_{\mu }\frac{p_{\mu }\;u_{\kappa }\left(p_{\mu }/\varepsilon \right)}{u_{\kappa }^{3}\left(p_{\mu }\right)}\;, \end{array}$$

and used the relation

(97) $$\begin{array}{c} \partial_{i}\gamma =\frac{1}{{\tilde{U}}_{\kappa }^{\;\;(1)}}\frac{p_{i}}{u_{\kappa }\left(p_{i}\right)}\equiv {\tilde{P}}_{i}^{\;(1)}\;, \end{array}$$

obtained from of the condition $\partial_{i}\left(\sum_{\mu }p_{\mu }\right)=0$.

Remarkably, in the escort formalism, the κ-Brègman divergence can be express in the language of the escort distribution according to

(98) $$\begin{array}{c} D\left[p,\;q\right]=E_{P}\left[\ln_{\kappa }\left(p\right)-\ln_{\kappa }\left(q\right)\right]\;, \end{array}$$

which has a very straightforward interpretation.

### 4.3. Third Statistical Structure

The last geometric structure that we are introducing came from the Brégman-like divergence and has been already studied from a different perspective in [68,69,70].

It is consistent with the following statistical model

(99) $$\begin{array}{c} S_{\left(3\right)}^{\kappa }=\left\{p\equiv \left\{p_{\mu }\left(\theta \right)\right\}:\;\Omega \to R\;\;|p_{i}=\frac{1}{\varepsilon }\;\exp_{\kappa }\left(\frac{1}{\lambda }\left(\theta^{i}-\gamma \left(\theta \right)\right)\right);\;p_{0}=1-\sum_{i}p_{i}\right\}\;, \end{array}$$

derived from the embedding

(100) $$\begin{array}{c} p_{\mu }\to \lambda \;\ln_{\kappa }\left(\varepsilon p_{\mu }\right)\;. \end{array}$$

To introduce a manifold structure on $S_{\left(3\right)}^{\kappa }$, let us identify the η-coordinate in $\eta \equiv \{p_{i}\}$ and apply relations (41) to $\rho \left[p,\;q\right]\equiv D_{\kappa }\left[q∥p\right]$. We obtain metric and connections as

(101) $$\begin{array}{c} g^{ij}=\lambda \;\frac{u_{\kappa }\left(\varepsilon \;\eta_{0}\right)}{\eta_{0}}\;+\lambda \;\frac{u_{\kappa }\left(\varepsilon \;\eta_{i}\right)}{\eta_{i}}\;\delta^{ij}\;, \end{array}$$

(102) $$\begin{array}{c} \Gamma^{ij,l}=\lambda^{2}\;\frac{u_{\kappa }\left(\eta_{0}\right)}{\eta_{0}^{2}}-\lambda^{2}\;\frac{u_{\kappa }\left(\eta_{i}\right)}{\eta_{i}^{2}}\;\delta^{ijl}\;, \end{array}$$

(103) $$\begin{array}{c} \Gamma^{*\;ij,l}=0\;. \end{array}$$

Clearly, $S_{\left(3\right)}^{\kappa }$ has a dually-flat structure compatible with the Hessian geometry related to the φ-potential $\varphi \left(\eta \right)\equiv -S_{\kappa }\left(\eta \right)$, that is the negentropy of $S_{\kappa }\left(\eta \right)$ given in Equation (9), so that the dual ∇-affine coordinate on Ξ are

(104) $$\begin{array}{c} \theta^{i}=-\frac{\partial S_{\kappa }\left(\eta \right)}{\partial \eta_{i}}\equiv \lambda \;\ln_{\kappa }\left(\varepsilon \;\eta_{i}\right)+\gamma \left(\theta \right)\;, \end{array}$$

while the ψ-potential follows from the Legendre transform on φ(η) and reads

(105) $$\begin{array}{c} \psi \left(\theta \right)=I_{\kappa }\left(\theta \right)+\gamma \left(\theta \right)\;. \end{array}$$

Remark that for positive measures, not constrained by $\sum_{\mu }p_{\mu }=1$, $I_{\kappa }$ is just the full Legendre transform of $S_{\kappa }$ in the probability space [68].

On the physical ground, the potential (105) corresponds to the κ-logarithm of partition function and is related to the free energy of the system according to

(106) $$\begin{array}{c} F_{\kappa }=-\frac{1}{\beta }\left(I_{\kappa }+\gamma \right)\;. \end{array}$$

Therefore, the third statistical structure, as well as the second one in its scaled formulation, reproduces and confirms the Legendre structure of the κ-deformed thermostatics previously derived by using standard arguments of statistical mechanics [35].

By using the expression of ψ(θ) it is ready to verify the first of Equations (34), that is

(107) $$\begin{array}{c} \eta_{i}=\partial_{i}\left(I_{\kappa }\left(\theta \right)+\gamma \left(\theta \right)\right)\;, \end{array}$$

so that metric and connections on the Ξ-space read:

(108) $$\begin{array}{c} g_{ij}=\frac{1}{\lambda }\frac{\eta_{i}}{u_{\kappa }\left(\varepsilon \;\eta_{i}\right)}\left(\delta_{ij}-\partial_{j}\gamma \right)\;, \end{array}$$

(109) $$\begin{array}{c} \Gamma_{ij,l}=0\;, \end{array}$$

(110) $$\begin{array}{c} \Gamma_{ij,l}^{*}=\frac{\eta_{i}}{u_{\kappa }\left(\varepsilon \;\eta_{i}\right)}\left[\frac{1}{\lambda }\frac{u_{\kappa }\left(\eta_{i}\right)}{u_{\kappa }^{2}\left(\varepsilon \;\eta_{i}\right)}\left(\delta_{ij}-\partial_{i}\gamma \right)\;\left(\delta_{il}-\partial_{j}\gamma \right)-\partial_{jl}\gamma \right]\;, \end{array}$$

that confirm the dually-flat structure of $S_{\left(3\right)}^{\kappa }$.

It is trivial to verify that canonical divergence D[p,q] on the statistical manifold $S_{\left(3\right)}^{\kappa }$ coincides, as expected, with the κ-Brègman divergence $D_{\kappa }\left[q∥p\right]$ according to [68]

(111) $$\begin{array}{c} D\left[p,\;q\right]=D_{\kappa }\left[q∥p\right]\;. \end{array}$$

Finally, let us just to observe that, in complete analogy with the discussion done in Section 4.2, the Hessian structure on $S_{\left(3\right)}^{\kappa }$ is also compatible with the scalar product

(112) $$\begin{array}{c} \langle A,\;B\rangle =E_{P^{\left(1\right)}}\left[A\;B\right]\;, \end{array}$$

where

(113) $$\begin{array}{c} E_{P^{\left(1\right)}}\left[f\right]=\sum_{\mu }P_{\mu }^{\left(1\right)}\;f^{\mu }\;, \end{array}$$

are the escort average and the κ-escort distribution $P^{\left(1\right)}$ is

(114) $$\begin{array}{c} P_{\mu }^{\left(1\right)}=\frac{1}{U_{\kappa }^{\left(1\right)}}\frac{p_{\mu }}{\lambda \;u_{\kappa }\left(\varepsilon \;p_{\mu }\right)}\;,\;\mathrm{with}\;U_{\kappa }^{\left(1\right)}=\sum_{\mu }\frac{p_{\mu }}{\lambda \;u_{\kappa }\left(\varepsilon \;p_{\mu }\right)}\;. \end{array}$$

Again, in the escort formalism, the κ-Brègman divergence can be written in the straightforward manner given by

(115) $$\begin{array}{c} D_{\kappa }\left[q∥p\right]=E_{P^{\left(1\right)}}\left[\ln_{\kappa }\left(p\right)-\ln_{\kappa }\left(q\right)\right]\;. \end{array}$$

## 5. Concluding Remarks

In the framework of the κ-formalism we have derived several geometric structures starting from non-equivalent κ-divergence functions corresponding, in the κ→0 limit, to the Kullback–Leibler, “Kerridge” and Brégman divergences, respectively.

The main results that characterize the emerging geometries, such as metric, connection, θ-coordinate and φ- and ψ-potentials, when available, are reported in the final Table 1, for an easy comparison.

In particular, the statistical manifold derived from the κ-Kullback–Leibler divergence has an invariant geometry with a dual structure and constant curvature. This geometric structure corresponds to the well-known α-geometry [1,2], with $\alpha =1+2\;\kappa^{2}$, and is related to the natural embedding of the standard logarithm.

The other two statistical manifolds, derived from the κ-Kerridge and κ-Brégman divergences, have a dually-flat structure with dual potentials related to each other by the corresponding Legendre transform. Their respective potentials are given by the scaled entropy (or parentropy) and scaled free energy in the first case and by the entropic form and free energy in the latter case. Both these geometries are compatible with an embedding based on the κ-logarithm and a scalar product based on the escort probability average. Moreover, the κ-Brégman functional turns out to be the natural canonical divergence in the own statistical manifold while the κ-Kerridge functional share with the canonical divergence the same cross term and therefore, it is equivalent to the corresponding canonical divergence. These dually-flat structures are together related by a merely scaling transformation of the potentials and the distributions. In this way, the structure based on the κ-Brégman functional has simplest expressions for the potentials with respect to the structure based on the κ-Kerridge functional that has a slimmer expression for the distribution.

## Figures and Tables

**Table 1:** Geometrical structures emerging from different κ-deformed versions of divergent function.

**κ-Kullback–Leibler**	$D\left[p,\;q\right]=\sum_{\mu }p_{\mu }\;\ln_{\kappa }\left(\frac{p_{\mu }}{q_{\mu }}\right)$
**metric**	$g^{ij}=\frac{1}{p_{0}}+\frac{\delta^{ij}}{p_{i}}$
**connection**	$\Gamma^{ij,l}=\left(1+\kappa^{2}\right)\;\left(\frac{1}{p_{0}^{2}}-\frac{\delta^{ijl}}{p_{i}^{2}}\right)$
**distribution**	$p_{i}=\frac{q_{i}}{\varepsilon }\;\exp_{\kappa }\left(\frac{1}{\lambda }\left({\tilde{\theta }}^{i}-\gamma \left(\tilde{\theta }\right)\right)\right)$
**$\tilde{\theta }$-coordinate**	${\tilde{\theta }}^{i}=\lambda \left(\ln_{\kappa }\left(\varepsilon \frac{p_{i}}{q_{i}}\right)+\ln_{\kappa }\left(\varepsilon \frac{p_{0}}{q_{0}}\right)\right)$
**θ-coordinate**	$\theta^{i}=\ln\left(p_{i}\right)-\ln\left(p_{0}\right)$
**ϕ-potential**	\ \
**ψ-potential**	\ \
**κ-Kerridge**	$D\left[p,\;q\right]=\sum_{\mu }p_{\mu }\;\left(\ln_{\kappa }\left(p_{\mu }\right)-\ln_{\kappa }\left(q_{\mu }\right)\right)$
**metric**	$g^{ij}=\frac{u_{\kappa }\left(\eta_{0}\right)}{\eta_{0}}\;+\frac{u_{\kappa }\left(\eta_{i}\right)}{\eta_{i}}\;\delta^{ij}$
**connection**	$\Gamma^{ij,l}=\lambda \;\frac{u_{\kappa }\left(\eta_{0}/\varepsilon \right)}{\eta_{0}^{2}}-\lambda \;\frac{u_{\kappa }\left(\eta_{i}/\varepsilon \right)}{\eta_{i}^{2}}\;\delta^{ijl}$
**distribution**	$p_{i}=\exp_{\kappa }\left(\theta^{i}-\gamma \left(\theta \right)\right)$
**θ-coordinate**	$\theta^{i}=\ln_{\kappa }\left(p_{i}\right)+\ln_{\kappa }\left(p_{0}\right)$
**ϕ-potential**	$\varphi \left(\eta \right)=-\frac{1}{\lambda }\;S_{\kappa }\left(\frac{\eta }{\varepsilon }\right)$
**ψ-potential**	$\psi \left(\theta \right)=\frac{1}{\lambda }\;{\tilde{I}}_{\kappa }\left(\theta \right)+\gamma \left(\theta \right)$
**κ-Brégman**	$D\left[p,\;q\right]=S_{\kappa }\left(q\right)-S_{\kappa }\left(p\right)-\lambda \sum_{\mu }\left(p_{\mu }-q_{\mu }\right)\ln_{\kappa }\left(\varepsilon \;q_{\mu }\right)$
**metric**	$g^{ij}=\lambda \;\frac{u_{\kappa }\left(\varepsilon \;\eta_{0}\right)}{\eta_{0}}\;+\lambda \;\frac{u_{\kappa }\left(\varepsilon \;\eta_{i}\right)}{\eta_{i}}\;\delta^{ij}$
**connection**	$\Gamma^{ij,l}=\lambda^{2}\;\frac{u_{\kappa }\left(\eta_{0}\right)}{\eta_{0}^{2}}-\lambda^{2}\;\frac{u_{\kappa }\left(\eta_{i}\right)}{\eta_{i}^{2}}\;\delta^{ijl}$
**distribution**	$p_{i}=\frac{1}{\varepsilon }\;\exp_{\kappa }\left(\frac{1}{\lambda }\left(\theta^{i}-\gamma \left(\theta \right)\right)\right)$
**θ-coordinate**	$\theta^{i}=\lambda \left(\ln_{\kappa }\left(\varepsilon \;p_{i}\right)+\ln_{\kappa }\left(\varepsilon \;p_{0}\right)\right)$
**ϕ-potential**	$\varphi \left(\eta \right)=-S_{\kappa }\left[\eta \right]$
**ψ-potential**	$\psi \left(\theta \right)=I_{\kappa }\left(\theta \right)+\gamma \left(\theta \right)$
